# Obstetricians’ Attitude towards Childbirth

**DOI:** 10.3390/ijerph182010650

**Published:** 2021-10-11

**Authors:** Ernesto González-Mesa, Jesús Jiménez-López, Marta Blasco-Alonso, Daniel Lubián-López

**Affiliations:** 1 Obstetrics and Gynecology Department, School of Medicine, University of Malaga, 29071 Malaga, Spain; 2 Grupo IBIMA de Investigación en Medicina Maternofetal, Epigenética, Enfermedades de la Mujer y Salud Reproductiva, IBIMA, 29071 Malaga, Spain; jesuss.jimenez.sspa@juntadeandalucia.es (J.J.-L.); martablal78@gmail.com (M.B.-A.); 3 Hospital Regional Universitario SSPA de Malaga, 29010 Malaga, Spain; 4 Department of Obstetrics and Gynecology, University Hospital Jerez de La Frontera, 11007 Cadiz, Spain; dmlulo@gmail.com; 5 Obstetric and Gynecology Department, Faculty of Medicine, Hospital Quirónsalud Campo de Gibraltar, University of Cadiz, 11007 Cadiz, Spain

**Keywords:** attitudes assessment, CAVE, childbirth humanization, birth-related trauma

## Abstract

(1) Background: In Spain, as in other countries, there is an increase in policies and practices focused on the humanization of perinatal care. In this regard, the quality of interpersonal interactions between women and health professionals is one of the main factors, and, apart from other factors, it is influenced by health professionals´ attitudes towards childbirth. The main objective of this study was to determine the attitudes of obstetricians towards the humanization of childbirth and the promotion of a positive childbirth experience. (2) Methods: The psychosocial task force of the Spanish Society of Obstetrics and Gynecology designed a nationwide online survey. The questionnaire on attitudes towards childbirth (CAVE, acronym for “cuestionario de actitudes sobre vivencias y experiencias en el parto”) was used for the assessment. Exploratory and confirmatory factor analyses of the scale were also performed. (3) Results: A total of 384 participants completed the survey. Obstetricians showed a high-quality clinical obstetric performance, but some difficulties in identifying birth-related psychological-trauma risk factors. Some differences according to practice and gender were found in the final score and in areas regarding psychosocial risk, pain, accompaniment, and women´s expectations. (4) Conclusions: In light of the results, it is advisable to launch education initiatives aimed to improve interaction with pregnant women.

## 1. Introduction

Unlike depression, post-traumatic stress disorder (PTSD) after childbirth is not routinely screened and goes largely unrecognized in maternity services [1]. Owing to the global prevalence of birth-related trauma, health professionals should be aware of its risk factors and symptoms to reduce the impact on the mental health of women and their offspring. Although birth-related trauma has been found in 20% to 48% of all deliveries [2], it is frequently overlooked in perinatal clinical practice, and among its risk factors, low-quality interactions with health care professionals have been reported to play an important role [3,4,5].

Pregnancy and childbirth are periods of potential psychological distress due to emotional, social, and economic adjustments. The promotion of women’s awareness about the effects of pregnancy on a mental disorder and an examination of their preferences and attitudes for care may reduce the sense of loneliness and mitigate anxiety or depressive symptoms [6]. Women’s feelings of being in control of the childbirth process depend largely on the physical and emotional support that professionals provide, especially when women have a history of obstetric psychological trauma [7]. Accordingly, health professionals should revise what specific aspects of their delivery-room practices could improve their interaction with women at childbirth, and promote a positive and humanized experience for women [8].

Humanizing childbirth involves fostering good clinical practices and promoting a positive culture based on dialogue, empathy, and respect [9]. To this end, during labor, professionals should provide sensitive clinical guidance, carry out a respectful assessment of an expectant mother’s anxieties regarding the whole process [10], take into account the uniqueness of each case, and perform procedures to improve the health of the mother and infant. All these improved practices should be incorporated into professional educational programs [11].

In Spain, as in other countries, policies and practices are being implemented to humanize perinatal care [12]. The quality policy of the Spanish Ministry of Health includes recommendations supported by the Spanish Society of Obstetrics and Gynecology (SEGO) to improve maternity care, facilitate the participation of women in decision-making during delivery, and support initiatives that could improve obstetric services in our hospitals [13,14]. However, some factors could hinder the humanization of childbirth in Spain, such as the difficulty that professionals sometimes can show in establishing the appropriate communicative interaction with expectant mothers [15]. Recently, the Committee on the Elimination of Discrimination against Women, in response to a lawsuit filed by a woman after childbirth, urged the Spanish government to promote research on issues regarding obstetric violence and to foster the training of health professionals on women’s reproductive health rights [16].

The main objective of this study was to determine the attitudes of Spanish obstetricians towards the humanization of childbirth and the promotion of a positive experience of childbirth.

## 2. Materials and Methods

### 2.1. The Survey

The Psychosocial Obstetrics and Gynecology section of the Spanish Society of Obstetrics and Gynecology (SGOP-SEGO) designed a nationwide online survey using its digital platform. The survey was conducted between October and November an invitation was sent by email to all partners, with a link to the questionnaire set. Three appeals were made at 10-day intervals. The SEGO currently has 5476 members, of which 1269 belong to its perinatal section, our target group.

The questionnaire included an informed consent document, a brief explanation of the reasons why the research was being held, the questionnaire on attitudes towards childbirth (CAVE, acronym for “cuestionario de actitudes sobre vivencias y experiencias en el parto”) [17,18], and a series of sociodemographic questions such as gender, age, city of residence and work, type of practice (public/private), and whether the participant had children. The survey was anonymized and included an authorization request for the subsequent analysis of the collected data. Ethical approval was previously obtained (PEYBA CAVE-001 1918-N-20).

### 2.2. Instrument

CAVE is a self-administered questionnaire developed to assess the attitude of health-science students towards the experiences of women during childbirth. The questionnaire consists of 52 Likert-type items whose scores range between 52 and 260, and where a favorable attitude would obtain the highest score. A standardized procedure for its development and validation included: item development by an international and multiprofessional group of academics and clinicians from different European countries (Spain, Iceland, Lithuania, Portugal, Turkey and the UK), psychometric pre-validation, Cronbach’s Alpha coefficient calculation, test–retest and item-total correlation for the reliability analysis. In addition, content validity was undertaken by a Delphi panel of 16 experts, over 2 rounds. The validation process has already been published [17], and the scale has been used in previous studies [18]. The Spanish language validation resource showed that the questionnaire was a valid and reliable tool, with a Cronbach´s alpha coefficient of 0. The questions were designed to assess different components of attitude, including reactions to women’s empowerment during labor, feelings about the accompaniment during childbirth, knowledge of the influence of psychosocial variables, professional role identity, reactions to possible unexpected outcomes, women’s expectations, management of clinical care priorities, knowledge of birth-related trauma (BRT), feelings on women’s participation in decision-making during labor, ‘medicalization’ of care, commitment, and respectful behavior [17].

### 2.3. Statistical Analysis

Cronbach’s alpha coefficient was used to measure the internal consistency of the questionnaire score. The Kaiser–Meyer–Olkin (KMO) test and Bartlett’s test of sphericity were performed to assess the adequacy of an exploratory factor analysis (EFA). An EFA was then conducted using the analysis of the main components of the scale, while the Varimax method and orthogonal rotation were used to identify latent factors that explained the observed variance. To determine the appropriate number of factors for the scale, the resulting eigenvalues (>1) were considered, and scree plots were examined.

To put a structure on the pattern of covariance between the first-order factors and to explain the covariance in a more parsimonious manner with fewer parameters, we hypothesized that some other higher-order factors accounted for the pattern of relationship between the first-order factors, performing a second-order EFA [19]. Subsequently, a confirmatory factor analysis was held, and structural equation modelling (SEM) analyses were performed with correlated factors, considering the maximum likelihood estimator. Four fit indices were selected to assess model fit: comparative fit index (CFI), Tucker–Lewis index, standardized root mean square (SRMS), and root mean square error of approximation (RMSEA). An acceptable model fit was defined by a CFI ≥ 0.90, Tucker–Lewis index ≥ 0.95, and SRMR, or RMSEA values ≤ 0.08 [20,21]. Based on these criteria, the best-fitting final model was selected. The statistical analysis was carried out using the SPSS program version 25.0 (IBM SPSS Statistics for Windows, Version 25.0, released 2018, IBM Corp., Armonk, NY, USA) and Stata program version 14.0 (StataCorp, Texas, LP, USA).

## 3. Results

The survey was completed by a total sample of 384 obstetricians from all regions of Spain. As shown in Table 1, most of the participants were female doctors aged between 35 and 50 years old. The sociodemographic features of the sample are shown in Table 1.

### 3.1. Exploratory Factor Analysis

**Figure 1:**
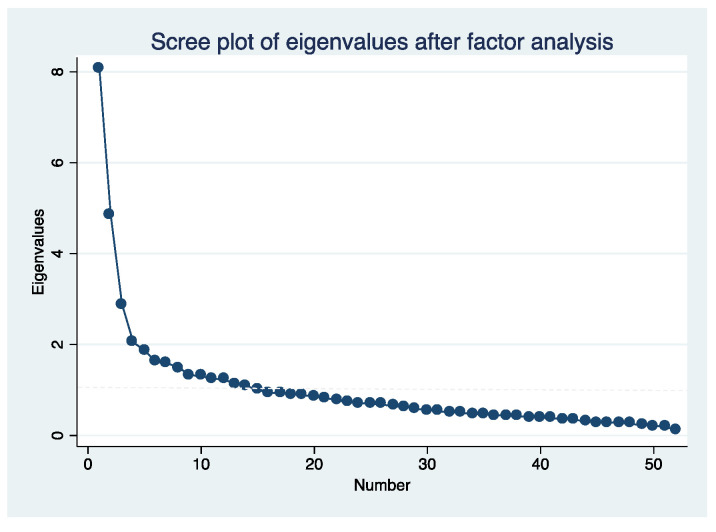
Scree plot for first-factor analysis.

**Figure 2:**
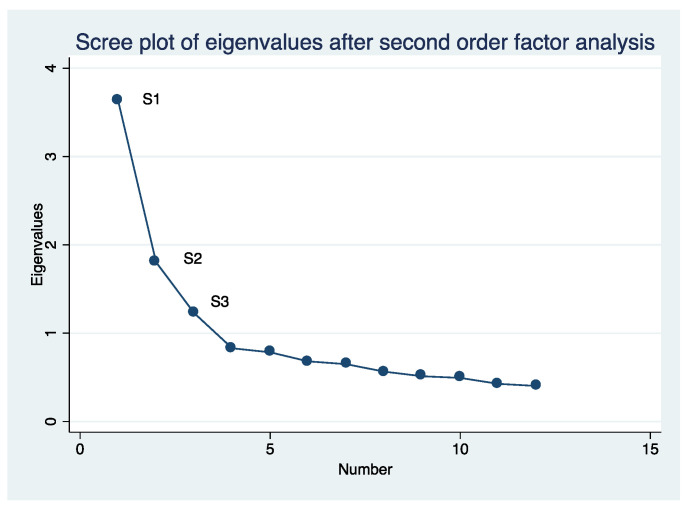
Scree plot for second-order factor analysis.

We found that Cronbach´s reliability coefficient was 0.883. We observed a KMO coefficient of 0.821, and Bartlett´s sphericity test showed a correlation between variables with a chi-square value of 6612.53, 1326 degrees of freedom, *p* < 0.0001. The EFA showed that 12 latent variables explained 63% of the total observed variance (Figure 1): F1, supremacy; F2, obstetric complications; F3, psychological risks; F4, pain management; F5, humanization; F6, women’s decision-making participation; F7, BRT aetiology; F8, psychosocial risk; F9, obstetric practice; F10, accompaniment; F11, commitment; F12, women’s expectations. The item distributions among the 12 factors with their saturation coefficients and the correlation coefficient among the factors are shown in Table 2 and Table 3, respectively. We carried out a second exploratory analysis on the referred first-order factors, similar to previous studies using CAVE in other populations [17]. The KMO coefficient was 0.81, and Bartlett’s sphericity test showed a chi-square value of 1003.33, with 66 degrees of freedom, and *p* < 0.0001. Our analysis revealed that three second-order factors explained 55.5 % of the total variance (Figure 2). The composition of the second-order factors and the saturation coefficients are listed in Table 4.

### 3.2. Confirmatory Analysis

**Figure 3:**
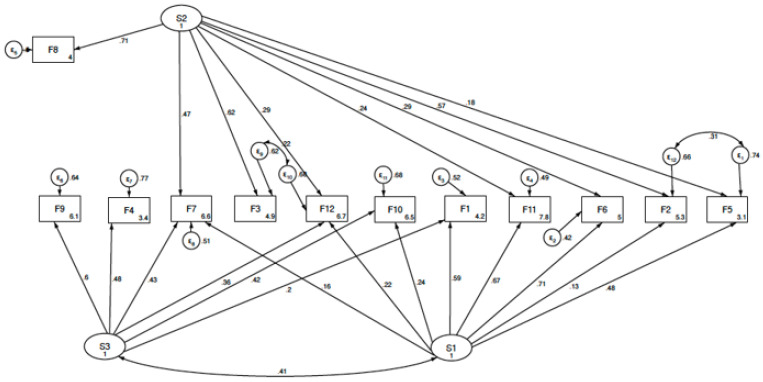
Second—order Factor Model F1, Supremacy; F2, Obstetric complications; F3, Psychological risks; F4, Pain management; F5, Humanization; F6, Women´s decision making; F7, Birth-related trauma etilology; F8, Psychosocial risk; F9, ObScheme; F10, Accompaniment; F11, Commitment; F12, Women´s expectations; S1, Ability to empathize; S2, BRT-risk management; S3, Obstetric performance.

Using structural equation models, we observed that the model with three sec-ond-order latent factors, i.e., ability to empathize (S1), BRT-risk factors (S2), and ob-stetric performance (S3), shown in Figure 3, presented better fit criteria than the model with four second-order factors, initially described in studies using CAVE in other pop-ulations. The fit indices for both models are shown in Table 5. S1 gathers information about interpersonal interaction. It explores the physicians´ thoughts about direct in-teraction with the women at childbirth, the need to ask for her consent to perform any intervention, to properly empathize with women and to identify themselves. It also explores their opinion about women’s expectations, the need for accompaniment, as well as the importance of attending to some other cultural or emotional needs. S2 as-sesses the knowledge and opinion about the issues that could prevent childbirth from becoming a positive experience, such as poor obstetric or neonatal outcomes, medical complications, women’s physical and mental health status, psychosocial risk factors, as well as the ability to identify potentially traumatic situations. S3 explores knowledge and beliefs about the medicalization of delivery care, and their ability to adequately prioritize care needs in the delivery room.

### 3.3. Scores

We observed a mean CAVE score of 193.21 (SD 21.19). The main descriptive statistics for CAVE and for the first- and second-order factors are shown in Table 6. The score distribution according to sociodemographic variables is shown in Table 7. We did not find any statistically significant difference in the score distributions according to the geographical origin of the respondents. However, we found significant differences according to the type of practice and gender of respondents. As shown in Table 7, the highest mean CAVE scores were found in cases of private practice and in female obstetricians. Regarding the information on maternity/paternity of the participants, we found significantly higher scores in the participants without children in factors F4 (pain management, 10.6; against 9.4; t 3.5; *p* < 0.01) and F7 (BRT aetiology; 21.7; against 20.6; t 2.8; *p* < 0.004). In contrast, participants with children scored significantly higher in the second-order factor S3 (obstetric performance, 6.4; against 5.9; t 2.5; *p* < 0.012).

## 4. Discussion

In this report, we described the validation process for the Spanish version of the CAVE questionnaire in a population of Spanish obstetricians to assess the attitudes of specialists towards women’s childbirth experiences. The CAVE questionnaire was made up of 52 Likert-type items that consisted of 12 latent components related to three major dimensions or second-order latent variables: the ability to empathize with women in labor, BRT-risk management, and obstetric performance.

Furthermore, we reported the results of a nationwide survey conducted by the SGOP-SEGO task force on the attitudes of Spanish obstetricians regarding the experience of childbirth. Our results indicated that although obstetricians scored moderate to high in all the dimensions, there is room for improvement in the current care for pregnant women, especially with respect to the quality of professional interaction and the management of psychosocial risks during childbirth. In these areas, the achieved scores were 68.2% and 78.3% of the maximum possible score, respectively.

The respondents showed some problems in establishing an empathic relationship with women during childbirth, with a bias in the decision-making process, as observed from the scores in factor S1. In this regard, no differences were found based on the type of practice, gender, or age of the specialists. S1 was related to interpersonal interaction, and to some possible distortions in the distribution of authority during childbirth, and the average score reached 71.3% of the maximum possible (Table 6).

Some studies have shown that in situations where the needs and demands of the patients do not fit the biomedical model of disease, the relationship becomes more difficult [22,23], and when childbearing women want care or decline care that is not aligned with the recommendations of their care provider, this can cause tension. During pregnancy and childbirth, values such as women’s emotional state, beliefs, expectations, cultural needs, sense of dignity and autonomy are important pillars for the psychological stability of women after delivery, but they are not always considered; some authority bias emerges. The reduction of this bias entails some measures such as encouraging patients to bring a list of their concerns and expectations to be previously discussed (the birth plan). In addition, the reduction of bias requires the recognition of and attention to the specific needs of disadvantaged groups [24], which means the recognition of psychosocial risk factors. Our results showed that the specialists have some problems in recognizing these psychosocial factors (F8), and therefore with the recognition of vulnerable women during childbirth (70% out of the maximum possible score). As many as 44.5% of the respondents showed displeasure when women presented a birth plan.

The exposure of specialists to stressful situations and the impact on their own mental health also need to be considered. Work-related PTSD may affect a specialist’s emotional reactions, resulting in a tendency to depersonalize the care recipients [25], a high rate of clinical interventions [26], and insensitive and defensive care [27], which negatively influence their social interactions.

Regarding BRT risk-factors management (S2), some studies [28,29] have reported that a lack of training in obstetric psychological trauma makes it more difficult to identify that, thus contributing to its persistence. Based on the results of the survey, we found that issues such as taking a woman’s opinion into account in the decision-making process during childbirth, prioritizing maternal and fetal needs in the delivery room, and avoiding unnecessary medicalization of labor are some of the goals that our perinatal educational program needs to address. Some progress has been made in this regard, and our results show that the new generations of specialists obtained higher scores in the second-order dimensions that CAVE evaluated (Table 7).

Finally, obstetric performance (S3) scores were high, but some structural and organizational changes in maternity wards have been claimed [12]. Maternity wards with women-centric structures in which continued perinatal care could be assured and where childbirth is considered a physiological process are necessary and would contribute to a more humanized obstetric practice, as reported in a recent study on midwives’ experiences in Catalonia (Spain) [30]. The lack of familiarity that specialists have with women in labor, when they treat them for the first time during childbirth, results in weak communication and a lack of reciprocal trust between pregnant women and professionals. Interpersonal relationships can be distorted in such situations, making obstetricians opt, almost automatically, for practices that they subjectively consider to be ‘safer’, even though the procedures might objectively be unnecessary [31].

Efforts should be made to foster women-centric maternity environments in which, together with pursuing the best possible obstetric and perinatal outcomes, women could feel in control of the situation, safe, and respected [32].

### Strengths and Limitations

This is the first survey carried out in Spain that focuses on the attitude of obstetricians towards the childbirth experience. The survey provides useful information on the aspects that need to be improved to enhance the humanization of perinatal care in Spain, promote a positive experience in women, and avoid obstetric traumas. SEGOP-SEGO addresses a specific continuous education program for its partners. Our work also presents the validation of a useful psychometric tool (CAVE). The survey analyses showed that it is a valid and reliable instrument to use in a professional context, in order to identify which areas of psychosocial care need to be improved in perinatal training programs for obstetricians or even to assess an obstetrician’s perception of risk in perinatal scenarios.

The main limitation of this study is related to the number of participants. Although the studied sample was representative of Spain’s obstetrician population, the response rate was only 30% of the target population. In addition, some selection bias may have occurred; the participating specialists might have been those who are most motivated on issues related to the humanization of perinatal care. Thus, our study may not fully reflect the reality of the country.

## 5. Conclusions

Through this study, we have proved CAVE to be a valid and reliable tool to study the attitude of obstetricians towards the childbirth experience. The scale is a self-administered tool with 52 items exploring three main factors: S1, ability to empathize; S2, BRT-risk factors; and S3, obstetric performance.

Participating specialists scored moderate to high in all the assessed dimensions, but some room for improvement can be recognized in the current attention to pregnant women, especially with respect to the quality of professional interaction and the management of psychosocial risks during childbirth, specifically PTSD risk factors. Considering these results, it is advisable to launch educational initiatives aimed at improving these matters in obstetricians, including communication skills, ethical education, and self-reflection.

## Figures and Tables

**Table 1:** Main sociodemographic features of the sample.

	*N* (%)
Age	
<35 years	104 (29.4)
35–50 years	128 (36.2)
>50 years	122 (34.5)
Gender	
Male	94 (26.6)
Female	260 (73.4)
Children	
No	105(29.7)
Yes	249 (70.3)
Practice	
Private	75 (21.2)
Public	181 (51.1)
Both	98 (27.7)
Community	
Andalucía	81 (22.9)
Aragón	16 (4.5)
Canarias	15 (4.2)
Cantabria	2 (0.6)
Castilla y León	12 (3.4)
Castilla-La Mancha	16 (4.5)
Cataluña	47 (13.3)
Comunidad de Madrid	59 (16.7)
Comunidad Valenciana	25 (7.1)
Extremadura	12 (3.4)
Galicia	25 (7.1)
Islas Baleares	9 (2.5)
La Rioja	1 (0.3)
Navarra	5 (1.4)
País Vasco	17 (4.8)
Principado de Asturias	4 (1.1)
Región de Murcia	8 (2.3)

**Table 2:** First-order exploratory factor analysis.

F1	F2	F3	F4	F5	F6	F7	F8	F9	F10	F11	F12
It 11 0.768	It 31 0.565	It 37 0.823	It 1 0.845	It 5 0.366	It 24 0.429	It 19 0.553	It 40 0.772	It 6 0.679	It 10 0.480	It 9 0.350	It 18 0.551
It 12 0.812	It 32 0.701	It 38 0.847	It 2 0.830	It 28 0.792	It 25 0.427	It 22 0.803	It 41 0.691	It 7 0.598	It 15 0.666	It 51 0.710	It 36 0.384
It 13 0.738	It 33 0.825	It 39 0.764	It 3 0.663	It 29 0.780	It 26 0.715	It 23 0.796	It 42 0.483	It 8 0.552	It 47 0.582	It 52 0.701	It 45 0.662
It 14 0.668	It 34 0.582			It 30 0.469	It 27 0.739	It 43 0.481			It 48 0.423	It 4 0.343	
It 50 0.468	It 35 0.747					It 44 0.576				It 49 0.719	
It 16 0.448										It 20 0.653	
It 17 0.446										It 21 0.630	
It 46 0.401											

First-order exploratory factor analysis; F1, Supremacy; F2, Obstetric complications; F3, Psycholog-ical risks; F4, Pain management; F5, Humanization; F6, Women´s decision making; F7, Birth-related trauma etilology; F8, Psychosocial risk; F9, Obstetric practice; F10, Accompaniment; F11, Com-mitment; F12, Women´s expectations.

**Table 3:** Correlation coefficients for first-order factors.

	F1	F2	F3	F4	F5	F6	F7	F8	F9	F10	F11	F12
F1	1.00											
F2	0.100	1.00										
F3	0.052	0.348	1.00									
F4	0.218	0.032	0.028	1.00								
F5	0.343 **	0.327 **	0.066	0.037	1.00							
F6	0.452 **	0.233 **	0.164 **	0.170 **	0.372 **	1.000						
F7	0.307 **	0.308 **	0.336 **	0.266 **	0.224 **	0.402 **	1.000					
F8	0.066	0.399**	0.431 **	0.009	0.100	0.198 **	0.320 **	1.000				
F9	0.267 **	0.035	0.004	0.254 **	0.173 **	0.197 **	0.295 **	0.051	1.000			
F10	0.380 **	0.096	0.051	0.295 **	0.204 **	0.315 **	0.315 **	0.027	0.338 **	1.000		
F11	0.459 **	0.233 **	0.151 **	0.141 **	0.347 **	0.534 **	0.374 **	0.190 **	0.191 **	0.298 **	1.000	
F12	0.234 **	0.203 **	0.314 **	0.231 **	0.204 **	0.330 **	0.389 **	0.179 **	0.303 **	0.265 **	0.274 **	1.000

Correlation coefficients for first-order factors; F1, Supremacy; F2, Obstetric complications; F3, Psychological risks; F4, Pain management; F5, Humanization; F6, Women´s decision making; F7, Birth-related trauma etilology; F8, Psychosocial risk; F9, Obstetric practice; F10, Accompaniment; F11, Commitment; F12, Women´s expectations; (marked with ** in case of statistical significance).

**Table 4:** Second order EFA; F1, supremacy; F2, obstetric complicactions; F3, Psychological risks; F4, Pain management; F5, Humanization; F6, women´s decision making; F7, birth-reñlated-trauma etilology; F8, Psychosocial risk; F9, Obstetric practice; F10, accompaniment; F11, commitment; F12, women´s expectations; S1, Ability to empathize; S2, BRT-risk management; S3, Obstetric performance.

S1 Empathy	S2 BRT Risk Management	S3 Obstetric Performance
F1	F2	F4
0.713	0.635	0.698
F5	F3	F9
0.717	0.801	0.706
F6	F7	F10
0.719	0.482	0.570
F11	F8	F12
0.723	0.770	0.509

**Table 5:** Structural equation models fitting índices.

	Chi-Square	df	RMSEA	CFI	AIC	BIC	SRMR	CD
Model 1 (four factors)	338.10	71	0.10 (0.09–0.11)	0.80	21,868.0	22,053.82	0.11	0.98
Model 2 (three factors)	370.42	41	0.00 (0.00–0.03)	1.00	204,520.9	206,420.58	0.02	0.97

Comparative fit index (CFI); Akaike’s information criterion (AIC); Bayesian information criterion (BIC); root mean square error of approximation (RMSEA); standardized root mean square (SRMS).

**Table 6:** Descriptive statistics for the scales.

Scale	25 th Percentile	50 th Percentile	75 th Percentile	Scale Rank	Mean	Std. Deviation	Scores Rank	Minimum	Maximum
CAVE	183	196	211	(52–260)	193.21	21.19	(52–260)	119.00	260.00
F1	24	29	34	(8–40)	28.64	6.81	(8–40)	8.00	40.00
F2	18	20	22	(5–25)	19.44	3.66	(5–25)	5.00	25.00
F3	10	12	14	(3–15)	11.79	2.42	(3–15)	3.00	15.00
F4	8	10	12	(3–15)	9.84	2.88	(3–15)	3.00	15.00
F5	7	9	11	(4–20)	9.39	3.07	(4–20)	4.00	20.00
F6	13	15	18	(4–20)	15.14	3.03	(4–20)	4.00	20.00
F7	19	22	23	(5–25)	20.96	3.19	(5–25)	7.00	25.00
F8	9	11	12	(3–15)	10.50	2.60	(3–15)	3.00	15.00
F9	12	14	15	(3–15)	12.95	2.11	(3–15)	3.00	15.00
F10	16	18	20	(4–20)	17.31	2.67	(4–20)	7.00	20.00
F11	26	29	31	(7–35)	28.55	3.68	(7–35)	12.00	35.00
F12	11	12	13	(3–15)	11.93	1.77	(3–15)	5.00	15.00
Second order factors									
S1	71	89	87	(23–115)	78.47	12.23	(23–115)	34.00	110.00
S2	57	63	69	(16–80)	62.69	8.54	(16–80)	34.00	80.00
S3	48	53	57	(13–65)	52.04	6.38	(13–65)	31.00	65.00

F1. supremacy; F2. obstetric complicactions; F3. Psychological risks; F4. Pain management; F5. Humanization; F6. women´s decision making; F7. birth-reñlated-trauma etilology; F8. Psychosocial risk; F9. Obstetric practice; F10. accompaniment; F11. commitment; F12. women´s expectations; S1 Ability to empathize; S2 BRT-risk management; S3 Obstetric performance.

**Table 7:** Scores distribution according to sociodemographic variables.

	CAVE	F1	F2	F3	F4	F5	F6	F7	F8	F9	F10	F11	F12	S1	S2	S3
Practice																
Private	200.52	28.04	19.40	11.10	9.08	8.97	14.86	19.92	10.20	12.49	16.17	28.41	11.56	77.10	60.62	49.30
Public	193.76	29.43	19.55	12.17	10.17	9.51	15.29	21.49	10.62	13.24	18.09	28.73	12.17	79.74	63.85	53.68
Both	196.47	27.63	19.25	11.62	9.81	9.48	15.06	20.77	10.48	12.75	16.75	28.31	11.79	77.18	62.14	51.12
F value	7.64	2.61	0.22	5.64	3.84	0.88	0.58	6.88	0.72	4.04	18.35	0.47	3.61	1.99	4.14	14.98
*p*	*p* ≤ 0.001	NS	NS	*p ≤* 0.004	*p ≤* 0.022	NS	NS	*p ≤* 0.001	NS	*p ≤* 0.018	*p ≤* 0.001	NS	*p ≤* 0.028	NS	*p ≤* 0.017	*p ≤* 0.001
Age																
<35 years	28.75	28.75	20.06	12.27	11.14	9.53	15.77	22.30	10.79	13.19	18.09	28.94	12.47	79.44	65.45	65.45
35–50 years	28.35	28.35	19.73	11.90	9.95	9.50	15.00	21.25	10.37	13.15	17.50	28.26	11.92	78.05	63.27	63.27
>50 years	28.84	28.84	18.59	11.27	8.61	9.14	14.74	19.50	10.37	12.53	16.45	28.51	11.50	78.09	59.74	59.74
F value	11.70	0.83	5.26	5.16	24.50	0.59	3.53	25.70	0.96	3.72	11.80	0.97	8.74	0.45	13.90	27.30
*p*	*p ≤* 0.001	NS	*p ≤* 0.006	*p ≤* 0.006	*p ≤* 0.001	NS	*p ≤* 0.03	*p ≤* 0.001	NS	*p ≤* 0.025	*p ≤* 0.001	NS	*p ≤* 0.001	NS	*p ≤* 0.001	*p ≤* 0.001
Gender																
Male	28.10	28.10	19.11	11.51	8.77	9.56	14.60	20.00	10.76	12.61	16.39	28.25	77.40	77.40	61.39	49.17
Female	28.83	28.83	19.55	11.90	10.22	9.33	15.33	21.30	10.40	13.07	17.65	28.65	78.86	78.86	63.16	53.08
t value	−2.89	−0.88	−0.99	−1.48	−4.27	0.63	−2.00	−3.45	1.15	−1.79	−3.57	−0.90	−3.58	−0.99	−1.73	−5.28
*p*	*p ≤* 0.004	NS	NS	NS	*p ≤* 0.001	NS	*p ≤* 0.046	*p ≤* 0.001	NS	NS	*p ≤* 0.001	NS	*p ≤* 0.001	NS	NS	*p ≤* 0.001

F1. supremacy; F2. obstetric complicactions; F3. Psychological risks; F4. Pain management; F5. Humanization; F6. women´s decision making; F7. birth-reñlated-trauma etilology; F8. Psychosocial risk; F9. Obstetric practice; F10. accompaniment; F11. commitment; F12. women´s expectations; S1 Ability to empathize; S2 BRT-risk management; S3 Obstetric performance; NS, not significant.

## Data Availability

All data files are available upon reasonable request to the corresponding author.
